# Refractory chronic urticaria in adults: clinical characterization and predictors of severity

**DOI:** 10.1186/s13223-020-00496-0

**Published:** 2020-11-11

**Authors:** Iolanda Alen Coutinho, Frederico Soares Regateiro, Rosa Anita Fernandes, Joana Sofia Pita, Raquel Gomes, Constança Coelho, Ana Todo Bom

**Affiliations:** 1 Allergy and Clinical Immunology Departement, Centro Hospitalar E Universitário de Coimbra, Coimbra, Portugal; 2 Institute of Immunology, Faculty of Medicine, University of Coimbra, Coimbra, Portugal; 3 ICBR - Coimbra Institute for Clinical and Biomedical Research, CIBB, Faculty of Medicine, University of Coimbra, Coimbra, Portugal; 4 Faculty of Medicine, University of Lisbon, Lisboa, Portugal; 5 Institute of Pathophysiology, Faculty of Medicine, University of Coimbra, Coimbra, Portugal

**Keywords:** Refractory chronic urticaria, Predictors of severity, Adults

## Abstract

**Background:**

Chronic urticaria (CU) is defined as recurrent urticaria lasting for more than 6 weeks.

**Objectives:**

We aimed to characterize the phenotypes of patients with CU refractory to standard dose anti-H1 antihistamine treatment and search for clinical predictors of poor disease control.

**Methods:**

Retrospective collection of data regarding clinical characteristics, comorbidities, treatment, and disease control of all adult refractory CU patients presenting to the Allergy and Immunology Department during 1 year.

**Results:**

Sixty-one adult patients were included, 74% females, average age 44.5 years (18 to 84 years old). Most patients (78.7%) had initiated CU less than 1 year before enrolment. Chronic spontaneous urticaria (CSU) accounted for 55.7% of the patients, CSU associated with chronic inducible urticaria (CIndU) as a comorbidity for 44.3%, and angioedema was present in 55.7%. Medically-confirmed psychiatric disorders were present in 78.7%. Complementary diagnostic tests were performed in cases with more severe presentation (UAS7 ≥ 28 and/or UCT < 12) or with longer evolution (> 1 year), corresponding to 42 tested patient. Evidence for autoimmunity (positive anti-thyroid peroxidase antibodies, anti-nuclear antibodies or autologous serum test) was found in 45.2% (n = 19/42), and high C-reactive protein was present in 14.3% (n = 6/42), half of these also had positive antinuclear antibodies. Forty-six patients (75.4%) had at least one significant exacerbation, requiring medical appointment, emergency room, hospitalization or job absenteeism. The number of exacerbations correlated with the presence of angioedema (p = 0.022), with a recent diagnosis (< 1 year), and with higher UAS7 severity (p = 0.006). Although ClndU was associated with poor symptom control (p = 0.022), it was also associated with less exacerbations requiring medical observation or hospitalization (p = 0.015). All patients were using antihistamines and 21.3% (n = 13) of them were also under treatment with omalizumab, ciclosporine or montelukast for disease control.

**Conclusions:**

Autoimmunity can affect about half of the patients with severe or long-term CU. UAS7 and angioedema are associated with disease exacerbations. UAS7 and UCT presented unequal accuracy, with UAS7 better associating with the occurrence of exacerbations and treatment doses. Patients with refractory CU frequently present psychiatric disorders. Accurate diagnostic tests, namely autoimmune parameters and inflammatory markers, should be recommended in some individual cases.

## Background

Urticaria is a mast-cell-driven disease characterized by the development of transient pruritic wheals with or without associated angioedema [1]. This definition excludes other medical conditions in which urticaria, angioedema or both may occur, such as anaphylaxis, auto-inflammatory syndromes, urticarial vasculitis (UV), or bradykinin-mediated angioedema, including hereditary angioedema [1].

Chronic urticaria (CU) is defined by the presence of recurrent urticaria, angioedema, or both, for a period of 6 weeks or longer [1, 2]. In most patients, CU is a self-limited disorder, with an average duration of 2 to 5 years. However, in up to 30% of the patients, the symptoms may persist for more than 5 years [1, 3].

CU is a common disorder, with a lifetime estimated prevalence of up to 1 percent of the general population in the United States, 7.8% in Germany, 9.0% in Southwest Norway and 3.4% in Portugal [1, 4–7]. CU is more common in adults than in children, and women are affected twice as often as men [8, 9]. The condition typically begins between the third and the fifth decades of life [4, 8].

The EAACI/GA^2^LEN/EDF/WAO consensus [1, 2] classified CU into two subtypes for clinical use: (1) chronic spontaneous urticaria (CSU), when no specific eliciting factors are identified, or (2) chronic inducible urticaria (ClndU), when specific stimuli trigger the symptoms. CIndU include symptomatic dermographism, cold urticaria, delayed pressure urticaria, solar urticaria, heat urticaria, vibratory angioedema, cholinergic urticaria, contact urticaria and aquagenic urticaria. Two or more different subtypes of urticaria may coexist in any given patient.

The diagnosis of CSU is based on clinical history, physical examination, and the evaluation of some specific factors that aggravate CSU in a substantial subset of patients, such as newly administrated drugs (e.g. nonsteroidal anti-inflammatory drugs—NSAIDS and hormonal therapies), infections (viral, bacterial and parasitic), IgE-mediated allergic reactions, insect stings, emotional stress, alcohol, and some dietary habits (e.g., spicy food) [9–18]. The diagnosis of CIndU is based on the patient's clinical history and, if possible, the result of provocation tests. In patients with suspected CIndUs, it is important to identify and accurately characterize the trigger to confirm diagnosis and assess disease activity.

Routine laboratory tests seldom reveal abnormalities when the medical history does not suggest an underlying allergic etiology or the presence of systemic disease [1, 19]. Complementary laboratory tests (erythrocyte sedimentation rate, C-reactive protein, blood count, complement factors, antinuclear antibodies, cryoglobulins, hepatitis B and C serologies, serum protein electrophoresis, thyroid function and others) are recommended only in cases in which the clinical history suggests an underlying etiology [1, 13, 20]. The concept that CU could represent an autoimmune disorder resulted from the recognition that thyroid dysfunction and thyroid autoantibodies are more prevalent in patients with CSU [21]. Antinuclear antibodies (ANAs) are also more prevalent in patients with CU than in the general population [8]. Consequently, the presence of autoantibodies can be investigated upon clinical suspicion by the autologous serum skin test (AutoST) or other markers such as ANAs or antithyroid antibodies [22]. Autoimmunity and autoallergy, due to IgG and/or IgE autoantibodies have been considered as an etiology of CU [23]. It was proposed that an autoantibody or other histamine release factor could be present in the serum of these patients (especially those who had positive autologous serum skin test), namely human IgG directed against the IgE receptor alpha subunit (anti-Fc-epsilon-R1-alpha) and human IgG directed against the region IgE Fc (anti-IgE) [24]. Although the presence of these autoantibodies can be demonstrated, their availability is still scarce, and, currently, its clinical utility is not well established. Skin biopsy may be performed to exclude UV, particularly in CU refractory to antihistaminic treatment, with individual painful lesions rather than pruritic, with purpuric lesions, or when systemic symptoms are present, such as arthralgias and/or fever [25]. Although UV is not currently part of the CU in current disease guidelines, its finding during CU evaluation can lead to a diagnosis of systemic diseases, mainly connective tissue diseases, such as systemic lupus erythematosus, Sjogren's syndrome, IgM paraproteinemia (Schnitzler Syndrome), serum disease, infections (hepatitis B, infectious mononucleosis), and hypersensitivity to drugs [26]. Histology findings can also predict the response to treatment. The presentation of a neutrophil pattern in histology has been associated with refractory chronic urticarial [27].

The EAACI/GA2LEN/EDF/WAO International Urticaria Directive recommends second generation H1-antihistamines (H1-AH) in standard dose therapy, however, treatment with H1-antihistamine leads to the absence symptoms in less than 50% of patients [1, 28]. The prospective study AWARE (Germany) analyzed 1550 patients with H1 anti-histamine-refractory CSU in a 1-year non-interventional trial. In this study, 59.1% of the patients had papules at least once in the last 6 months, 16.1% reported at least 1 episode of angioedema, and 28.2% had moderate/high/very high impact on quality of life, namely due to pruritus, sleep disturbance and mental status disorders [28]. Regarding control medications, 17.4% of the patients were not following guideline recommended CU treatment [29]. A similar AWARE study was conducted in Portugal and included 76 patients [30]. It showed that both wheals and angioedema independently affect chronic urticaria quality of life questionnaire (CU-QoL) [30]. Guideline recommended non-sedative H1-AH treatment was used in almost 91.0% of patients at enrollment [30]. A total of 43.9% had moderate to severe urticaria, out of which 35.4% were medicated with third line therapy (omalizumab or cyclosporine), while 10.8% used oral corticosteroids, a lower percentage compared to the study performed in Germany [29].

There is a growing need for standardization of biomarkers to assess the severity and activity of CU and improve response to treatment. The objectives of our study were: (1) to characterize the clinical features, subtypes, cofactors, treatment and disease control status in adult refractory CU patients followed in an Allergy and Immunology Department (2) and to look for clinical and laboratorial predictors of poor disease control (including both exacerbations and symptom control).

## Methods

This is a retrospective, cross-sectional, and inferential study that included all adult patients with medically‐confirmed diagnosis of CU (as defined by EAACI guideline [1, 2]) refractory to approved doses of H1‐AH treatment, that were observed at the outpatient clinic of the Allergy and Immunology Department of Centro Hospitalar e Universitário de Coimbra, Portugal, during one year.

### Disease characteristics outcomes

Patients were evaluated and characterized according to demographic data, subtypes of urticaria, angioedema, associated comorbidities, such as medically diagnosed psychiatric disorders. Asthma and other atopic diseases were also evaluated according to European Academy of Allergy and Clinical Immunology and Global Initiative for Asthma criteria. Diagnostic work-up and treatment data were collected from the medical records at the same time as CU control was evaluated, using two methods:

1. Significant exacerbations—defined as CU exacerbations that required unscheduled medical consultations, emergency room admission, hospitalization or job absenteeism during the previous year;
2. Activity and disease control scores: Urticaria Control Test (UCT) [31] and Urticaria Activity Score 7 (UAS7) [32].

### Statistical analysis

Statistical analysis was performed using SPSS Statistics version 24.0®. Descriptive statistics were analyzed as mean and standard deviation for the variables with normal distribution, and median and interquartile range for the variables without normal distribution. Variables were also described in absolute number (n). The nominal variables were compared using Pearson’s chi-square test or Fisher's exact test according to Cochran’s rules. The normal distribution of the ordinal variables was evaluated using the Kolmogorov–Smirnov test (considering a population sample of more than 30 individuals in both groups). The comparison of these variables was tested using Student's T-tests (parametric test, applied after verifying the homogeneity of variances by the Levene test) or Mann–Whitney test (non-parametric test). A Type I error of 0.05 was considered.

## Results

### Demographics and CU clinical characteristics

Sixty-one adult patients with CU were evaluated, 73.8% (n = 45) were female and 26.2% (n = 16) male. The median age of patients was 44.5 ± 14.9 years, ranging from 18 to 84 years old. Most patients (78.7%) had initiated CU symptoms less than 1 year before enrollment (Table 1).

**Table 1:** Demographics and medical history of the patients included

Variable	Population cohort (n = 61)
Age	
Median, years (min, max)	44.5 (18, 84)
Gender	
Women	73.8% (n = 45)
Years since urticaria diagnosis	
< 1 year	78.7% (n = 48)
2–5 years	9.8% (n = 6)
6–10 years	8.2% (n = 5)
> 10 years	3.2% (n = 2)
Comorbidities	57.4% (n = 31)
Allergic diseases	
Asthma and rhinitis	32.8% (n = 20)
Rhinitis	29.5% (n = 18)
Asthma	21.3% (n = 13)
Food allergy	3.3% (n = 2)
Atopic dermatitis	1.64% (n = 1)
Autoimmune diseases	
Autoimmune thyroid disease	11.5% (n = 7)
Psoriatic arthritis	1.64% (n = 1)
Cardiometabolic	
Arterial hypertension	19.7% (n = 12)
Diabetes	6.6% (n = 4)
Obesity	4.9% (n = 3)
History of acute myocardial infarction	1.64% (n = 1)
Psychiatric disease	
Depression and/or anxiety disorder	78.7% (n = 48)
Malignant diseases	
Follicular thyroid tumor	1.64% (n = 1)

Please note that some patients present several comorbidities

Regarding the characterization of the CU type, 55.7% (n = 34) were diagnosed with CSU and 44.3% (n = 27) with CSU associated with CIndU as a comorbidity. The ClndU subtypes diagnosed in our population are shown in Table 2 (70.4% of patients were afflicted by more than on type of ClndU and all had associated spontaneous urticaria). Females accounted for 88.9% (n = 24/27) of ClndU patients, while only 61.8% of the CSU (n = 21 /34) were females (p = 0.021, Chi-Square Tests).

**Table 2:** ClndU subtypes in the study population

ClndU subtypes	Number of patients
Delayed pressure urticaria	19.7% (n = 12)
Cholinergic urticaria	14.8% (n = 9)
Heat urticaria	14.8% (n = 9)
Symptomatic dermographism	13.1% (n = 8)
Cold urticaria	4.9% (n = 3)
Contact urticaria	4.9% (n = 3)
Solar urticaria	1.6% (n = 1)
Aquagenic urticaria	1.6% (n = 1)

Please note that some patients present with several CIndU subtypes

Thirty-four patients (55.7%) had at least one episode of angioedema within the last year. The presence of angioedema did not significantly associate with sex, age, recent onset of CU or the subtype of CU.

### Comorbidities

The frequency of comorbid atopic diseases was: asthma in 21.3% (n = 13), rhinitis in 29.5% (n = 18) and the combination of the two diseases in 32.8% (n = 20). Medically‐confirmed psychiatric disorders (depression and/or anxiety disorder) were present in 78.7% (n = 48). Other comorbidities, such as arterial hypertension, type 2 diabetes and obesity were present in 31.2% (n = 19).

### Diagnostic work-up

Complementary diagnostic tests were performed in cases in which the clinical history suggested an underlying etiology, according to the local follow-up protocols, namely for patients with more severe presentation (UAS7 ≥ 28 and/or UCT < 12) or with longer evolution (> 1 year).

Complementary tests performed for further classifying CU were complete blood count (CBC), erythrocyte sedimentation rate (ESR) and C-reactive protein (CRP), all performed in 68.9% (n = 42), and the least frequent tests were skin biopsy, requested for only 6 patients. All patients evaluated had a CBC within the reference values; CRP was high in six patients (6/42 = 14.3%) and half of these also had positive ANAs. In patients with high CRP results, infectious causes such as HSV, HBV, CMV, EBV and HIV were excluded. Regarding ESR testing (n = 42), none of the tested patients presented abnormal values (1–20 mm/h).

Evidence for autoimmunity markers (positive anti-thyroid peroxidase antibodies, anti-nuclear antibodies or AutoST) was found in 45.2% (n = 19) of 42 tested patients. ANAs assay was positive in one third (n = 14) of 42 tested patients. The “dense fine speckled” was the most frequent (n = 10), followed by the “nucleolar pattern” (n = 4). Routine measurement of thyroid stimulating hormone (TSH) has not been approved by international guidelines since 2018 [1]. Among patients who had positive ANAs, 21.4% (n = 3/14) had a diagnosis of autoimmune thyroid disease (AITD). The remaining 4 cases of AITD were ANAs negative, corresponding to a total of seven patients (7/42 = 16.7%).

The AutoST was performed in 9.8% (n = 8) patients with suspected diagnosis of autoimmune urticaria. Only one patient (out of 8) had a positive AutoST. This patient had been diagnosed with CU less than 1 year before evaluation and had a severe presentation of the disease, only partially controlled with the four antihistamines/day and omalizumab, and with frequent job absenteeism due to CU. The patient was also positive ANAs but negative for autoimmune thyroid antibodies.

Skin biopsy was performed on patients with unusual presentations, namely painful lesions, corresponding to a total of 9.8% (n = 6) patients; histological findings in all cases were compatible with the diagnosis of urticaria, showing interstitial edema with mixed perivascular infiltrate (lymphocytes, eosinophils and a few neutrophils/basophils).

### Treatment

All patients were on first-line therapy with non-sedating H1 antihistamines at the time of evaluation, with the majority of the patients receiving a twice-a-day regimen (52.4%, n = 32, Table 3). Among those treated with omalizumab (n = 4), four had CSU, one of them with autoimmune urticarial confirmed by positive AutoST, and one had several subtypes of ClndU, namely cholinergic urticaria, pressure urticaria and symptomatic dermographism. Atopy was present in all patients receiving montelukast therapy as additional therapy, resulting in an improvement of CU control with its introduction and in a reduction in the number of daily antihistamines to 1 per day in all cases. One patient was treated with ciclosporin after therapeutic failure with omalizumab. This was a 45-year-old female patient, with obesity and arterial hypertension, with CSU associated with angioedema diagnosis < 1 year and presenting poorly controlled CU and angioedema despite medication with cetirizine four times a day (40 mg) and showing no clinical worsening of arterial hypertension at the time of data collection.

**Table 3:** Active treatment at enrolment in the study population

Variable	Population cohort (n = 61)
Treatment	
Non-sedative H1-antihistamines	100% (n = 61)
Number of daily doses	
4	14.8% (n = 9)
3	13.1% (n = 8)
2	52.4% (n = 32)
1	19.7% (n = 12)
Montelukast	
Yes	13.1% (n = 8)
Omalizumab	
Yes	6.6% (n = 4)
Systemic corticotherapy	
Yes	6.6% (n = 4)
Ciclosporine	
Yes	1.6% (n = 1)

Three patients were evaluated during acute exacerbations and were under treatment with systemic corticosteroid therapy as additional therapy.

### Clinical exacerbations and disease control

Disease status was evaluated using two outcomes: significant exacerbations and activity/disease control scores (UAS7 and UCT). Significant exacerbations of CU were defined by the need of unscheduled medical consultations, emergency room, hospitalization or job absenteeism. Forty -six patients (75.4%) had at least one significant exacerbation during the previous year. The median number of visits to the Emergency Department (ED) was one visit/patient/year, with the majority of patients (45.9%, n = 28) presenting with one or two ED episodes in one year, and a maximum value of four visits in one year. Hospitalizations due to CU exacerbation occurred in 8.2% (n = 5) of the patients, and four of these patients had both CU and angioedema presentation for less than 1 year. Unplanned consultations were needed in about half of the population and job absenteeism occurred in 14.8% (n = 9) of patients, with a maximum of 90 days and a minimum of 1 day (Table 4).

**Table 4:** CU exacerbations (ED episodes, hospitalizations, unplanned consultations and job absenteeism)

Variable	Population cohort (n = 61)
CU exacerbations	***75.4% (n = 46)***
Visits to the emergency department	
> 3	9.8% (n = 6)
1–2	45.9% (n = 28)
0	44.3% (n = 27)
Hospitalizations	
2	3.3% (n = 2)
1	4.9% (n = 3)
0	91.8% (n = 56)
Unplanned consultations	
> 7	4.9% (n = 3)
5–7	8.2% (n = 5)
3–4	19.7% (n = 12)
1–2	19.7% (n = 12)
0	47.5% (n = 29 =)
Job absenteeism	
Yes	14.8% (n = 9)

The number of exacerbations correlated with higher UAS7 symptom scores (p = 0.006, Spearman correlation). In our sample, patients with ClndU had worst disease control scores (UAS7 and UCT questionnaires) when compared to patients with CSU only (p = 0.022, Mann–Whitney test). However, ClndU patients had fewer exacerbations requiring medical observation or hospitalization (p = 0.015, Mann–Whitney test).

A high number of antihistamines, use of corticosteroid therapy and/or ciclosporin for disease control correlated with higher UAS7 scores (p = 0.006, Spearman correlation) but no significant correlation was observed with the UCT score. The presence of angioedema associated with a higher number of exacerbations (p = 0.022, Mann–Whitney test) but with no differences concerning disease control (UAS7 and UCT), or the number of antihistamines used. Atopy (allergic asthma, allergic rhinitis, food allergy and atopic dermatitis) and autoimmunity (positive antithyroid peroxidase antibodies, ANAs and/or AutoST) did not significantly associate with the symptom scores or exacerbations. No other statistically significant associations were observed in relation to other variables, namely, complementary diagnostic tests or the presence of comorbidities.

## Discussion

We here presented new data on refractory CU and looked for clinical factors associating with worse outcomes (exacerbations and symptom control).

This study reflects the resources allocated to diagnose and treat patients with refractory chronic urticaria and describes the real-life scenario and clinical management in an urticaria center in a tertiary hospital. Similar to other studies in Portugal and other countries [29, 30, 33, 34], more than 70% of the patients were female and the median age was around 45 years old. CSU represented more than 55% of all cases of CU, however, a wide variability has been found [34–36]. Although the predominance of female patients was evident in the two groups of CU, CSU and ClndU, patients with ClndU had a higher predominance of females compared to CSU. This does not corroborate the literature, which indicates the absence of significant gender differences in ClndU compared to other types of urticarial [37].

Although provocation tests should be performed whenever possible, the risk of serious reactions during provocation tests results in low application of these tests [2]. However, in our center, patients with suspected CIndUs are tested and remain under the supervision of a doctor and nurse for a specific time, according to the suspected trigger. In the specific case of delayed pressure urticaria, the medical supervision time at our center is 6 h after the test is performed. In cases where symptoms appear later, we encourage patients to make a photographic record. This justifies that despite delayed pressure urticaria is found to be a relatively rare form of physical urticaria in most case series [38], in our study it was present in a high number of patients (44.4% of ClndU, n = 12/27). It appears immediately (within minutes) or, more commonly, 4 to 6 h after a variety of pressure stimuli, which is not consistently recognised by the patients and clinicians. In our clinic, we routinely inquire the specific signs of the different triggers for all types of inducible urticaria, which is followed by the specific test to confirm the diagnosis.

Being a mast cell disease and potentially associated with an autoimmune disease, diagnoses of systemic autoimmune diseases and other mast cell disorders should be considered [39, 40]. Apparently, CU is associated with a specific pattern of comorbidities, including autoimmune, atopic and psychiatric diseases and there is no clear pattern in relation to malignant and cardiovascular diseases [41]. In a Danish study by *Ghazanfar *et al. which evaluated the comorbidities of patients with CU in all centers in Denmark, over a 21-year period, atopic diseases were strongly overrepresented among patients [41]. In our population, about one third of patients had at least one associated atopic disease. *Ghazanfar *et al. also found a high prevalence anxiety/depression in the group of patients with CU, which is in line with the results of our work [40]. Hypertension has been associated with a prolonged duration of CU [42], although other studies that examined the prevalence of cardiovascular diseases in populations with CU did not find an increased risk of these comorbidities compared to the general population [43]. Our work showed a percentage of about 20.0% of patients who had a previous diagnosis of arterial hypertension. In this subgroup, with the exception of one patient who experienced therapeutic failure with omalizumab, the choice of treatment with cyclosporine was not considered by clinicians, speculating the consideration that its presence influenced the therapeutic choice. It is known that corticosteroids influence the glycemic profile, so it did not seem surprising that patients with a diagnosis of diabetes were not on corticosteroid therapy. Skin biopsy was performed on 9.8% of the patients. This procedure is not routinely needed for the diagnosis of CU but should be considered in newly formed wheal if urticarial vasculitis is suspected.

The use of 2nd generation H1 antihistamines was observed in the whole sample, as recommended by current guidelines [1], contrarily to other studies in which the therapy was not totally in agreement with those suggested by the guidelines [29]. The number of patients undergoing third-line therapy with omalizumab, ciclosporine and montelukast was 21.3% (n = 13), which differed from other series, with a higher value [29].

CBC, ESR and CRP were requested for most patients, which is in line with European and recommendations [1]. Autoimmunity has been implicated in CU, resulting in the activation of systemic inflammatory processes that have implications in the evolution and control of the disease. High levels of CRP have been related to the presence of a systemic inflammatory process of autoimmunity in CU and, therefore, to disease activity [44]. Takahagi et al. demonstrated that high levels of CRP correlated with disease activity [45]. Our work also found the presence of a group of patients with high CRP values, half of which also had positive antinuclear antibodies, demonstrating the importance of evaluating this parameter as a possible predictor of poor control and relationship with autominuity.

Autoimmune urticaria is implicated as a cause of CU in about 30% to 50% of idiopathic cases [46]. In autoimmune uricaria, the involvement of autoantibodies causing histamine release after reaction with immunoglobulin E (IgE) epitopes or with the high affinity IgE receptor chain (Fc RI) receivers, should be considered, especially in positive AutoST [1, 46]^.^ Due to the lack of access to these methodologies in our hospital at the time of data collection, this complementary method had not yet been carried out.

ANAs are a group of autoantibodies directed against nucleus antigens and are found in many patients with autoimmune diseases. These autoimmunity markers are positive in patients with CU by approximately 15–29% [42, 46].The detection of ANAs in serum has been carried out for several years to screening for autoimmune diseases, but this analysis lacks some degree of subjectivity. While it may be important to acknowledge the presence of these autoimmune markers to understand the CU mechanism, its role is not fully explained, neither its research criteria fully defined. Margen et al. published a study aimed at identifying clinical and laboratory attributes of patients with positive ANAs and CU [46]. One of the conclusions of the study was that this subgroup of patients (with positive ANAs) was characterized by greater refractoriness to treatment with standard licensed doses of antihistamines, despite the fact that most patients do not have associated autoimmune diseases [45]. These results were consistent taking into account the small prevalence of autoimmune diseases in the general population (around 5–7%) [45]. Our work showed an important percentage of positive ANAs patients, however only a few cases were diagnosed with autoimmune diseases. These results speculate the need to standardize the measurement of these markers in all population with CU refractory to antihistamine therapy, reinforcing the need to avoid their arbitrary research.

Thyroid disease is the most commonly reported autoimmune disease in patients with CU [46]. Approximately 5 to 34% of patients with CU have antithyroid antibodies and another 5 to 10% have clinically or biochemically confirmed thyroid disease [46]. The association between AITD and CU is an unsolved mystery and the criteria for when to request this type of complementary study are not completely defined/clarified [47]. Thus, the fact that not all patients in our population have undergone this complementary study is justified.

It is known that the prevalence of psychiatric disorders are evident in CU, however there are limited studies on their association with CU severity [48]. The present study in patients with refractory CU showed an overwhelming prevalence of psychiatric disorders (depression and/or anxiety), highlighting the potential for predicting poor disease control and the need for a multidisciplinary approach in these patients. The high prevalence can also be justified by the fact that there is an important prevalence of these disorders in the Portuguese general population, according to the World Health Organization in a 2015 data survey [49], where Portugal had an estimated prevalence of 5.7% for depression and 4.9% for anxiety. These comorbidities should not be neglected in patients with chronic diseases such as CU, with research being of crucial importance.

Although the level of evidence for the efficacy of treatment with leukotriene receptor anatgonists being low, the drug that has been showing better results was montelukast [50]. It was found that at the data collection, patients who were treated with montelukast had atopy (asthma and/or rhinitis with positive tests for aeroallergens) and CU disease control.

In our sample, the use of severity and therapeutic monitoring scores—UAS7 and UCT—presented unequal accuracy, with UAS7 better associating with the occurrence of exacerbations and treatment doses, emphasizing the recommended routine use of the UAS7 score has an important therapeutic value for the daily monitoring of patients. It is important to highlight that angioedema is not part of the UAS7 questionnaire. Patients with exacerbations requiring unplanned consultations, emergency visits or hospitalization were mainly patients with a shorter course of disease (< 1 year). According to the literature, ClndU treatment is mainly symptomatic with avoidance of eliciting triggers [37]. Thus, most of our exacerbating population also corresponds to patients with CSU whose trigger has not been identified, resulting in a more challenging symptom control.

Regarding our data, it appears that the presence of angioedema may indicate a possible predictor of poor disease control, making the choice of treatment crucial for its control. Sussman et al. considered that the presence of angioedema in CU is underreported and that it seems to be associated with an important negative impact, namely in the quality of life in daily activities and work performance, compared with patients who have CU without angioedema [51].

## Conclusions

This study involved a limited number of patients that were regularly observed at an Allergology and Clinical Immunology Department of a University Hospital. It was decided to make a multidisciplinary approach involving also psychiatrists and Internal Medicine specialists to better characterize refractory chronic urticaria. Patients were also submitted to a large number of complementary diagnostic tests to identify associated pathology. Despite having some limitations, namely in its retrospective nature, several conclusions could be drawn in our work: (1) CSU alone is frequent but CSU associated with CIndU as a comorbidity can affect almost half of the patients; (2) The CSU symptomatic group with more exacerbations can include patients with a recent diagnosis (< 1 year); (3) Angioedema can often be present and be correlated with an higher number of exacerbations; (4) UAS7 and UCT presented unequal accuracy, with UAS7 better associating with the occurrence of exacerbations and treatment doses; (5) Patients with refractory CU can be prone to psychiatric disorders, such as depression and/or anxiety and (6) accurate diagnostic tests, namely autoimmune parameters and inflammatory markers, should be recommended in some individual cases.

It is important to have a growing knowledge and a uniform management of refractory CU with its characterization by phenotypes, which make it possible to identify predictors of therapeutic response in order to contribute for better and personalized strategies to control this disease.

## Data Availability

Not applicable.

## Abbreviations

AITD: Autoimmune thyroid disease
ANA: Anti-nuclear antibody
AutoST: Autologous serum test
CBC: Complete blood count
CMV: Cytomegalovirus infection
CRP: C-reactive protein
CU: Chronic urticaria
CSU: Chronic spontaneous urticaria
ClndU: Chronic inducible urticarial
EBV: Epstein–Barr virus infection
ED: Emergency department
HBV: Hepatitis B infection
HIV: Human immunodeficiency virus infection
HSV: Herpes simplex virus infection
NSAIDS: Nonsteroidal anti-inflammatory drugs
VU: Vasculitic urticaria
